# Evaluating dose delivered of a behavioral intervention for childhood obesity prevention: a secondary analysis

**DOI:** 10.1186/s12889-020-09020-w

**Published:** 2020-06-08

**Authors:** William J. Heerman, Evan C. Sommer, Ally Qi, Laura E. Burgess, Stephanie J. Mitchell, Lauren R. Samuels, Nina C. Martin, Shari L. Barkin

**Affiliations:** 1 Department of Pediatrics, Vanderbilt University Medical Center, 2146 Belcourt Ave., Nashville, TN 37212-3504 USA; 2 Department of Biostatistics, Vanderbilt University Medical Center, 2525 West End Ave., Nashville, TN 37203-1741 USA; 3 Department of Psychology and Human Development, Vanderbilt University, 230 Appleton Place, Nashville, TN 37203-5721 USA

**Keywords:** Childhood obesity, Behavioral interventions, Dose intensity

## Abstract

**Background:**

Current recommendations for intensive behavioral interventions for childhood obesity treatment do not account for variable participant attendance, optimal duration of the intervention, mode of delivery (phone vs. face-to-face), or address obesity prevention among young children. A secondary analysis of an active one-year behavioral intervention for childhood obesity prevention was conducted to test how “dose delivered” was associated with body mass index z-score (BMI-Z) across 3 years of follow-up.

**Methods:**

Parent-child pairs were eligible if they qualified for government assistance and spoke English or Spanish. Children were between three and 5 years old and were at risk for but not yet obese (BMI percentiles ≥50th and < 95th). The intended intervention dose was 18 h over 3-months via 12 face-to-face “intensive sessions” (90 min each) and 6.75 h over the next 9 months via 9 “maintenance phone calls” (45 min each). Ordinary least-squares multivariable regression was utilized to test for associations between dose delivered and child BMI-Z immediately after the 1-year intervention, and at 2-, and 3-year follow-up, including participants who were initially randomized to the control group as having “zero” dose.

**Results:**

Among 610 parent-child pairs (intervention *n* = 304, control *n* = 306), mean child age was 4.3 (SD = 0.9) years and 51.8% were female. Mean dose delivered was 10.9 (SD = 2.5) of 12 intensive sessions and 7.7 (SD = 2.4) of 9 maintenance calls. Multivariable linear regression models indicated statistically significant associations of intensive face-to-face contacts (B = -0.011; 95% CI [− 0.021, − 0.001]; *p* = 0.029) and maintenance calls (B = -0.015; 95% CI [− 0.026, − 0.004]; *p* = 0.006) with lower BMI-Z immediately following the 1-year intervention. Their interaction was also significant (*p* = 0.04), such that parent-child pairs who received higher numbers of *both* face-to-face intensive sessions (> 6) and maintenance calls (> 8) were predicted to have lower BMI-Z. Sustained impacts were not statistically significant at 2- or 3-year follow-up.

**Conclusions:**

In a behavioral intervention for childhood obesity prevention, the combination of a modest dose of face-to-face sessions (> 6 h over 3 months) with sustained maintenance calls (> 8 calls over 9 months) was associated with improved BMI-Z at 1-year for underserved preschool aged children, but sustained impacts were not statistically significant at 2 or 3 year follow-up.

**Clinical trial registration:**

The trial was registered on ClinicalTrials.gov (NCT01316653) on March 16, 2011, which was prior to participant enrollment.

## Background

Many trials designed to prevent or treat childhood obesity have shown only modest and unsustained effects on child weight [1–6]. One possible explanation for this inconsistency is the variability in the dose of the intervention, which is commonly described by two parameters—contact time (i.e., “how much”, which is typically measured in hours) and duration (i.e., “how long”, which is typically measured in months) [7]. The U.S. Preventive Services Task Force (USPSTF) has recently recommended that lifestyle-based interventions for the treatment of obesity among children involve at least 26 contact hours, based on an assessment that interventions with fewer hours are less likely to be successful [8]. However, the authors of the USPSTF recommendations highlight that it is unclear whether the 26-h recommendation will be relevant in settings with inconsistent participant adherence, in interventions for young children, or in an obesity prevention context.

The implications of these uncertainties were highlighted by a recent systematic review and meta-regression that found that the dose of a behavioral intervention was unrelated to effect size on child weight outcomes [9]. The relationship between dose and weight-related outcomes is unclear partially because of variability in how behavioral intervention dose is categorized and quantified [10, 11]. The NIH Treatment Fidelity Framework distinguishes between “how an intervention was intended to be delivered” vs. “how well providers adhere to the intended treatment, including information about actual dose and content delivered” [12]. This suggests that it is important to assess the intervention dose actually received by each participant (i.e., “dose delivered”) as opposed to what was intended or assigned (i.e., “dose intended”) [1, 13]. Despite recommendations to measure dose delivered, most behavioral interventions in childhood obesity limit their process evaluation to dose intended [9]. Consequently, there is limited evidence to quantify the appropriate dose or duration required to support obesity prevention for underserved populations at higher risk for the emergence of childhood obesity.

The purpose of this study was to test the extent to which dose delivered during a recently completed behavioral childhood obesity prevention randomized trial (The Growing Right Onto Wellness Trial) was associated with childhood weight outcomes. We hypothesized that a higher number of individual-level intervention contacts, would be associated with lower child BMI-Z at 1, 2, and 3-year follow-up.

## Methods

In a post-hoc analysis of the Growing Right Onto Wellness Trial (GROW), we evaluated the relationship between dose delivered and child body mass index Z-score (BMI-Z) at multiple follow-up timepoints. GROW was a randomized controlled trial (RCT) of a parent-child intervention designed to prevent childhood obesity. Complete methods of GROW have been previously published [14]. The primary outcome of the trial was child BMI trajectory over a 3-year study; intention-to-treat analyses found no clinically meaningful or statistically significant differences between the trajectories in the intervention and control groups at 36 months [15]. Study procedures were approved by the Institutional Review Board of Vanderbilt University Medical Center (IRB No. 120643). All participants signed informed consent prior to participation in their language of choice (English or Spanish) [16, 17]. The trial is registered at ClinicalTrials.gov (NCT01316653).

### Participants

Parent-preschool child pairs were recruited from Davidson County, Tennessee. Participants were recruited from zip code regions proximal to two collaborating community recreation centers. Pairs were eligible to participate if they were eligible for government assistance (e.g., Supplemental Nutrition Assistance Program [SNAP], Special Supplemental Nutrition Program for Women, Infants, and Children [WIC]), spoke English or Spanish, the parent was over 18 years old, the child was between the ages of three and five, and both parent and child could participate in physical activity. We enrolled children with BMI percentiles ≥50th and < 95th defined by CDC standardized growth curves to reach those most at risk but who were not yet obese [18].

### Intervention

**Fig. 1:**
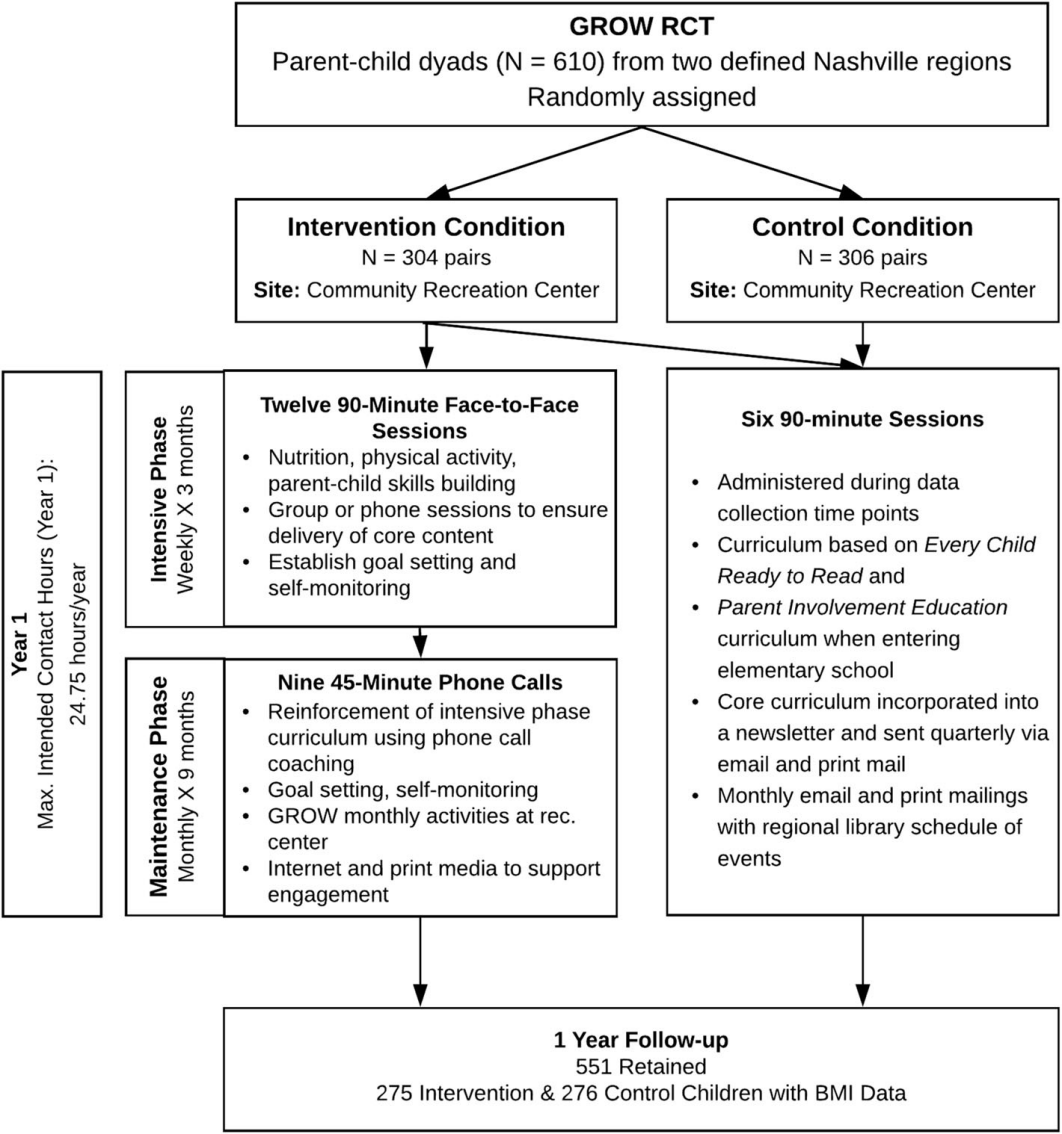
Study design of the GROW trial, indicating intended dose and data collection time points. At 12-month follow-up, 90.4% (275/304) of participants were retained in the intervention condition and 90.2% (276/306) of participants were retained in the control condition

The intended dose of the intervention (Fig. 1) included a maximum of 18 contact hours across the initial 3-month Intensive phase (90-min/week for 12-weeks, face-to-face group setting delivery) and 6.75 contact hours across the subsequent 9-month Maintenance phase (45-min/month for 9-months comprised of individual monthly phone call coaching), for a total maximum of 24.75 h in the first year [14, 15]. The intensive phase included two modes of delivery: face-to-face sessions or alternative sessions. Face-to-face sessions were facilitated by a trained interventionist in small groups at the community recreation center and typically lasted 90 min. Sessions were delivered in English or Spanish based on participant preference. If participants missed a session or knew they could not attend a pre-scheduled 90-min session, interventionists delivered alternative sessions as a shorter one-on-one phone call or individualized in-person session (typically lasting 20–30 min). The 12-weekly sessions focused on topics such as nutrition, physical activity, and parent-child skills-building. The 9-month maintenance phase included monthly coaching phone calls focusing on goal setting, self-monitoring, and problem-solving around key content areas. Fidelity to the intervention curriculum was measured using standardized protocols and was > 99% across all phases of the intervention.

Intervention and control groups received control content, which included a 45-min session on school readiness/success during four data collection time points, monthly mailings with a library schedule of events, and quarterly newsletters.

### Study procedures

Community liaisons (e.g., local pastors) helped recruit participants from community sites serving the target population. Demographics and other self-reported measures were collected by guided verbal administration of a survey. Certified data collectors measured child and parent height and weight to calculate baseline BMI.

### Measures

Dose delivered was measured by attendance recorded on sign-in sheets and verified by interventionists at intensive phase face-to-face sessions and electronic process evaluation data recorded by interventionists for all phone call sessions.

The primary outcome for this analysis was child BMI-Z at 1-year follow-up (i.e., immediately following the 1-year intervention), which was collected following the completion of the intensive and maintenance phases (i.e., the active intervention phases). Secondary outcomes include two- and three-year BMI-Z collected as a part of the original trial, following a passive intervention phase (texts and monthly mailings) where no active intervention dose was delivered. BMI-Z is based on child height, weight, age, and gender and was calculated using reference data available from the 2000 CDC growth charts for children [18]. Child height was measured to the nearest 0.1 cm using wall-mounted stadiometers, and weight was measured to the nearest 0.1 kg using research-grade, calibrated scales.

Potential confounders were identified based on possible associations with both childhood obesity and intervention participation. Variables included: baseline child age, gender, BMI-Z, Healthy Eating Index (HEI), [19] moderate and vigorous physical activity (MVPA), household SNAP or WIC utilization, parent race/ethnicity, baseline parent age, depression, stress, education level, and obesity status, parent classification of child weight, two “energy to change behavior” survey items, and four “confidence in ability to change behavior” survey items.

Psychosocial and sociodemographic characteristics were measured through parent self-report and were selected for the current analysis based on the conceptual model underlying the intervention [14]. Parent-reported child diet was assessed through 24-h diet recalls using Nutrition Data System for Research software. Diet recall data were used to calculate the 2010 HEI score for all children with two to three diet recalls (at least one weekday and one weekend day) completed within a 45-day window [19]. All participants were invited to complete dietary recalls. Days on which dietary recalls were attempted were randomly chosen and completion of recalls was often dependent on participant availability. Of the three recalls conducted, at least one recall was conducted more than 7 days after the initial recall. For the current analysis, 66.8% of children completed all 3 dietary recalls and 33.2% completed 2 dietary recalls. Child physical activity was assessed through accelerometers. Children were asked to wear a tri-axial GT3X+ accelerometer on their waist for 24-h a day on seven consecutive days to assess total amount and patterns of physical activity. Cut points based on previously published algorithms were used to assess percent of wear time spent in moderate and vigorous physical activity (MVPA) for children who met the minimum wear time criteria [20]. The two “energy to change” survey items were self-reported parent energy required to change their child’s 1) eating and 2) physical activity behaviors, and the four “confidence in ability to change” survey items were 1) confidence that their child would succeed in achieving healthy growth, and confidence that their family would be able to make changes to their 2) eating, 3) physical activity, and 4) media use. Each of these items was measured on a 10-point Likert-type scale with high values indicating more energy required for change or greater confidence in ability to change. Parent depressive symptoms were measured using the Center for Epidemiologic Studies Depression Scale (CES-D) and parent stress was measured using the perceived stress scale [21–24].

### Statistical analysis

Univariate statistics were used to describe dose, sociodemographic variables, anthropometric measures, and measures of child diet and accelerometry.

Ordinary least-squares multivariable regression was utilized to test for associations between dose delivered and child BMI-Z. Separate models were conducted for each dose modality (i.e., the number of intensive phase face-to-face contacts received, and the number of maintenance phone calls completed). Sessions received by alternative delivery were not included as face-to-face contacts in the analyses.

The interactive effect of the two dose modalities was tested by adding their main effects and their interaction to two separate multivariable models to facilitate interpretation. The first model utilized child BMI-Z as the outcome. The second model utilized adjusted logistic regression to examine how dose might predict the probability of achieving at least a 0.1 decrease in BMI-Z. The cutoff was set slightly below the suggested range of clinically meaningful BMI reduction for children 6 years and older (0.15–0.2) identified by the USPSTF [8, 25] to serve as a more sensitive threshold for potentially important BMI change in the younger sample analyzed in this study. Finally, a multivariable linear regression analysis was conducted to identify covariates that might predict dose received within the intervention group.

All models adjusted for baseline child BMI-Z, baseline child age, child gender, and parent race/ethnicity. Control participants had values of zero for all types of intervention dose and were included in each analysis (except for the model predicting dose received within the intervention group). However, sensitivity analyses were also conducted, limiting the analytic sample to those randomized to the intervention. Regression coefficients with 95% confidence intervals (CI) are presented along with graphical output to illustrate model-estimated predictive margins or contour plots for selected models. The assumption of linearity between dose and outcome was examined through distributional diagnostic plots of the residuals as well as by conducting regression models with restricted cubic splines and testing for nonlinearity [26]. Because interpretation of diagnostic plots and nonlinearity tests agreed that departure from linearity was not substantial for the primary analyses, we report only the linear model results. For the analyses evaluating the association between participant characteristics and the dose received, distributional assumptions were not met. As such, we report those models using robust standard errors.

While children in the intervention were nested within small subgroups at each of the two recreation centers, almost all of the outcome variance was at the child-level. There was no detectable improvement in model fit by adding a clustering level to the model, and multilevel model results were practically identical to single-level results. Because of this, and to preserve parsimony, all results presented are from single-level models.

All analyses were conducted using Stata version 14.2.

## Results

### Participant demographics

Of the 2126 families assessed for eligibility, 610 were randomized, with 304 assigned to the intervention group and 306 to the control group (Fig. 1). Among the 610 parent-child pairs randomized at baseline, the mean child age was 4.3 (SD = 0.9) years, and 316 (51.8%) child participants were female. The mean parent age was 32.1 (SD = 6.0), and 589 (96.6%) parent participants were mothers. The majority of parents self-identified as Hispanic (556, 91.1%); 39 (6.4%) of parents self-identified as Black, non-Hispanic. The majority of reporting households (530, 87.5%) received SNAP or WIC services. Participant baseline characteristics and BMI-Z at 1, 2, and 3-year follow-up are shown by dose received in Table 1.

**Table 1:** Participant Characteristics and BMI-Z

	Zero dose^a^ (*N* = 306)	Low dose^a^ (*N* = 134)	High dose^a^ (*N* = 170)	Total (*N* = 610)
Parent age (years)	31.6 (5.8)	32.1 (6.5)	32.9 (5.9)	32.1 (6.0)
Parent ethnicity				
Hispanic Mexican	204 (66.7%)	71 (53.0%)	112 (65.9%)	387 (63.4%)
Hispanic non-Mexican	74 (24.2%)	50 (37.3%)	45 (26.5%)	169 (27.7%)
Non-Hispanic	28 (9.2%)	13 (9.7%)	13 (7.6%)	54 (8.9%)
Parent education				
Less than high school	192 (62.7%)	81 (60.4%)	101 (59.4%)	374 (61.3%)
High school or more	114 (37.3%)	53 (39.6%)	69 (40.6%)	236 (38.7%)
Parent obesity status				
No	185 (60.5%)	73 (54.5%)	103 (60.6%)	361 (59.2%)
Yes	121 (39.5%)	61 (45.5%)	67 (39.4%)	249 (40.8%)
WIC and/or SNAP use				
No	31 (10.2%)	19 (14.4%)	26 (15.3%)	76 (12.5%)
Yes	273 (89.8%)	113 (85.6%)	144 (84.7%)	530 (87.5%)
N	304	132	170	606
Child age (years)	4.3 (0.9)	4.4 (0.9)	4.2 (0.9)	4.3 (0.9)
Child gender				
Male	144 (47.1%)	65 (48.5%)	85 (50.0%)	294 (48.2%)
Female	162 (52.9%)	69 (51.5%)	85 (50.0%)	316 (51.8%)
Child BMI-Z at baseline	0.8 (0.5)	0.8 (0.5)	0.8 (0.5)	0.8 (0.5)
Child BMI-Z at 1-year follow-up	0.9 (0.7)	0.9 (0.7)	0.8 (0.7)	0.8 (0.7)
N	275	109	165	549
Child BMI-Z at 2-year follow-up	1.0 (0.9)	1.1 (0.8)	0.9 (0.8)	1.0 (0.8)
N	266	112	166	544
Child BMI-Z at 3-year follow-up	1.3 (1.0)	1.4 (1.1)	1.2 (1.0)	1.3 (1.0)
N	272	111	165	548

^a^ Dose is intensive face-to-face sessions combined with maintenance calls (range: 0–21). Low dose is defined as less than the median number of sessions or calls (1–15) and high dose is defined as the median or more (16–21)

### Distribution of dose delivered

The majority of intervention participants (216, 71.1%) received all 12 intensive phase sessions via a combination of face-to-face intensive sessions and alternative intensive sessions. The mean number of weekly face-to-face sessions attended per parent-child pair was 7.2 (SD = 3.7), or 10.7 (SD = 5.5) hours, on average each session was 1.5 h (Table 2). In the maintenance phase, 229 (75.3%) received at least 80% (8 to 9 sessions or 6 to 6.75 h) of the scheduled monthly phone call coaching dose. When combining overall number of contacts between intensive weekly sessions (either modality) and monthly maintenance calls, 253 (83.2%) of intervention participants received at least 80% (17 to 21) of the intended dose for the one-year active phase of the behavioral intervention (Additional file 1).

**Table 2:** Distribution of Dose Delivered in the Intervention Group (*n* = 304). The intended dose of the intensive phase was 12 weekly sessions, completed either by a face-to-face session or an alternative session (e.g., phone call). The intended dose of the maintenance phase was 9 monthly phone calls. Dose delivered is presented as the mean (standard deviation) number of sessions each parent-child pair received. Participants in the control group (*n* = 306) received zero dose and their data is not included in this table

	Dose Intended	Mean Dose Delivered	Approximate Contact Hours^**a**^
Intensive Face-to-Face Sessions	12 Weekly Sessions	7.2 (3.7)	10.7 (5.5)
Intensive Alternative Sessions		3.8 (3.0)	1.6 (1.3)
Total Intensive Sessions		10.9 (2.5)	18.1 (5.6)
Total Maintenance Phone Calls	9 Monthly Phone Calls	7.7 (2.4)	5.8 (1.8)

^a^ Approximate contact hours calculated based on the following assumptions: intensive face-to-face sessions were 1.5 h, intensive alternative sessions were 0.42 h (25 min), and maintenance phone calls were 0.75 h. Approximate contact hours for total intensive sessions is based on the preceding assumptions as applied to the particular combination of face-to-face and alternative sessions completed by each individual participant pair

### Distribution of BMI-Z

By design, BMI-Z at baseline was limited in range, with a mean of 0.8 (SD = 0.5) [14]. At 1-year follow-up, 549/610 (90%) of children had sufficient data for analysis. The mean child BMI-Z was 0.8 (SD = 0.7, *n* = 549) at 1-year follow up. Immediately following the 1-year intervention, 61.4% (*n* = 337/549) of children were normal weight (i.e., BMI <85th percentile), 26.0% (*n* = 143/549) of children were overweight (BMI ≥85th percentile and < 95th percentile), and 12.6% (*n* = 69/549) of children were obese (BMI ≥95th percentile) based on standardized growth curves published by the U.S. Centers for Disease Control and Prevention [27].

### Fully adjusted associations between dose and BMI-Z

The multivariable linear regression model demonstrated a statistically significant main effect indicating that higher attendance at weekly face-to-face intensive sessions was associated with lower BMI-Z immediately following the 1-year intervention (B = -0.011; 95% CI [− 0.021, − 0.001]; *p* = 0.029). This result suggests that for every additional face-to-face session attended, BMI-Z at 1 year was reduced by 0.011. In addition, a child who attended the mean number of face-to-face sessions (7.2 sessions) would be estimated to have a BMI-Z that is 0.09 units lower than a similar child with 0 sessions. The corresponding model for number of monthly maintenance calls received demonstrated a similar main effect (B = -0.015; 95% CI [− 0.026, − 0.004]; *p* = 0.006). This result suggests that for every additional maintenance session attended, BMI-Z at 1 year was reduced by 0.015. In addition, a child with the mean maintenance session attendance (7.7 sessions), would be estimated to have a BMI-Z that is 0.116 units lower than a similar child with 0 sessions. Results of the full models for BMI-Z at 1 year are shown in Additional file 2. At 2- and 3-year follow-up, these associations followed the same general pattern but were smaller in magnitude and no longer statistically significant at the 5% level (2-year follow-up: face-to-face B = -0.008; 95% CI [− 0.021, 0.005]; *p* = 0.231; maintenance B = -0.006; 95% CI [− 0.020, 0.008]; *p* = 0.385. 3-year follow-up: face-to-face B = -0.009; 95% CI [− 0.025, 0.006]; *p* = 0.240; maintenance B = -0.010; 95% CI [− 0.026, 0.007]; *p* = 0.266). Results of the full models for BMI-Z at 2 and 3 years are shown in Additional file 3. Model-based estimates for each combination of dose received are shown in Additional file 4.

**Fig. 2:**
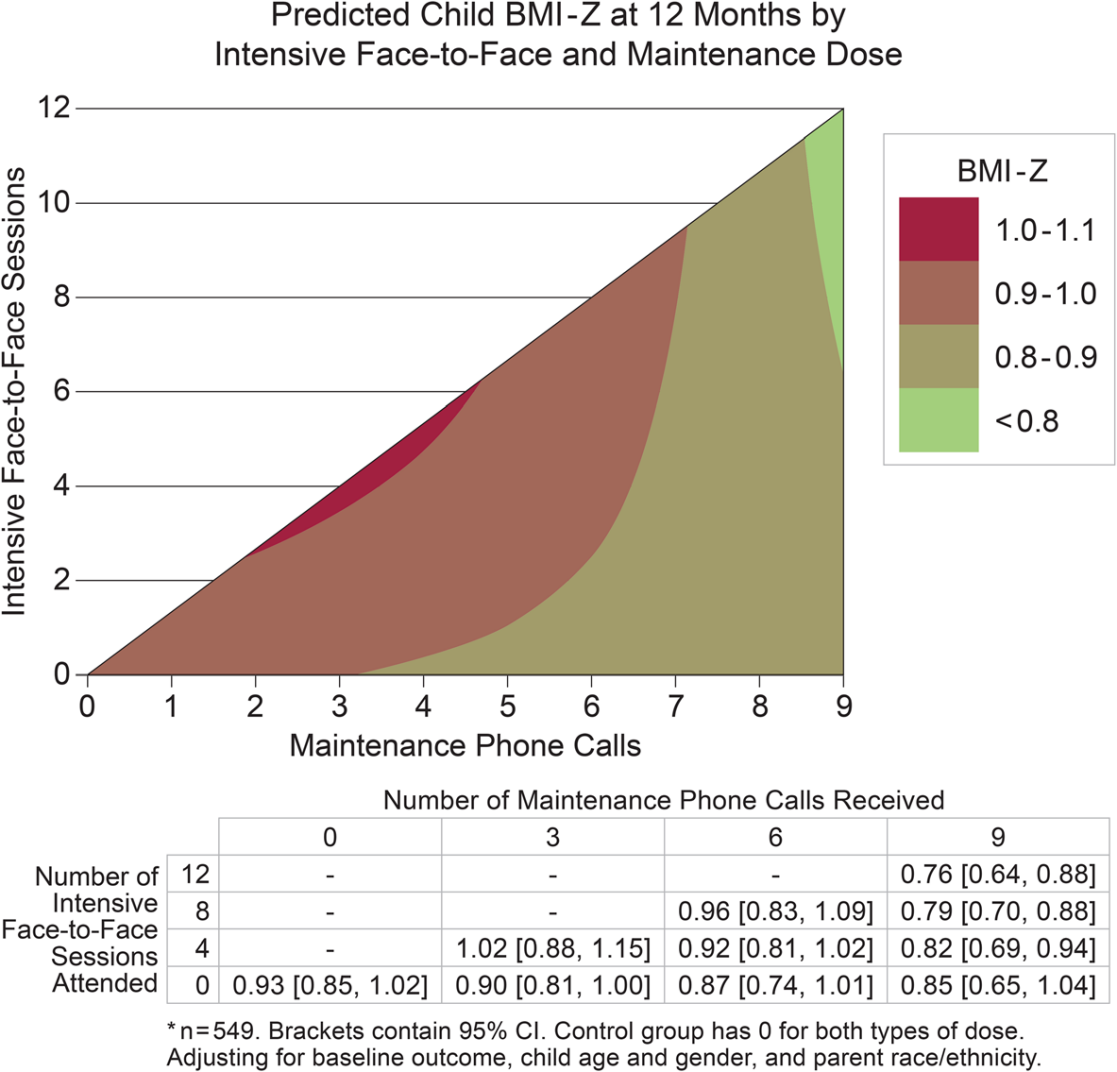
Contour plot of model-based estimates of child BMI-Z score immediately following the 1-year intervention. Children with high levels of both intensive face-to-face and maintenance phone calls had the lowest predicted BMI-Z. The data table shows predicted BMI-Z values for representative combinations of intensive and maintenance dose. This model included the main effects of face-to-face dose, maintenance dose, and their interaction, controlling for baseline child BMI-Z, child age, child gender, and parent race/ethnicity. To estimate predicted values, the following covariate profile was selected: males with the mean baseline BMI-Z, mean baseline age, who had parents of Hispanic, Mexican origin. Models using a variety of other covariate profiles generated similar results. Predicted estimates are not shown when beyond the bounds of the dose combinations present in the data (e.g., combinations of many face-to-face sessions and few maintenance phone calls). See Additional file 1 for complete distribution of dose received and additional file 5 for predicted estimates at each specific dose combination

**Fig. 3:**
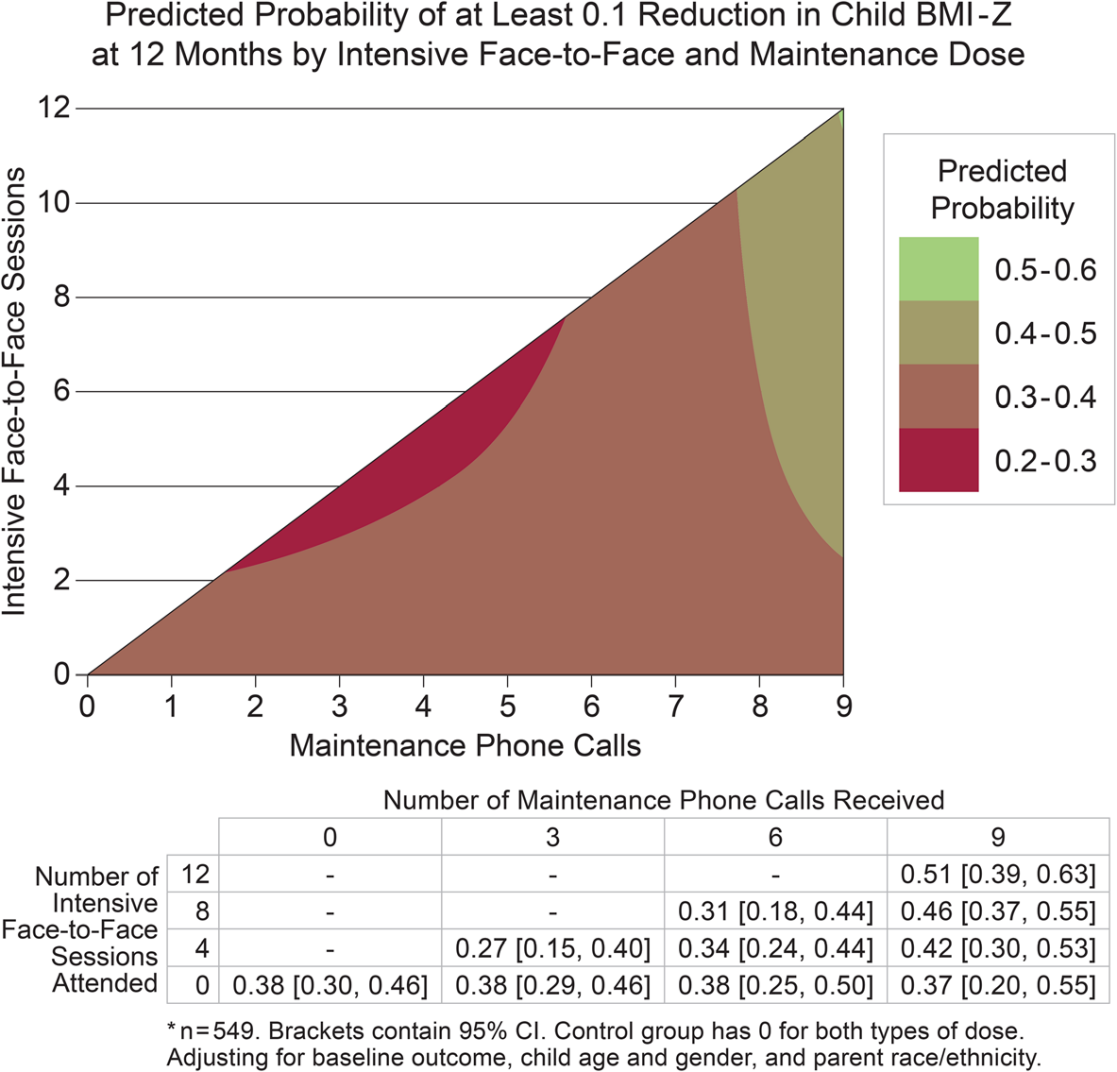
Contour plot of model-based estimates for the probability of at least a 0.1 decrease in BMI-Z immediately following the 1-year intervention. Children with high levels of both intensive face-to-face and maintenance phone calls had the highest probability of decreasing BMI-Z immediately following the 1-year intervention. The data table shows predicted probabilities for representative combinations of intensive and maintenance dose. This model included the main effects of face-to-face dose, maintenance dose, and their interaction, controlling for baseline child BMI-Z, child age, child gender, and parent race/ethnicity. To estimate predicted values, the following covariate profile was selected: males with the mean baseline BMI-Z, mean baseline age, who had parents of Hispanic, Mexican origin. Models using a variety of other covariate profiles generated similar results. Predicted estimates are not shown when beyond the bounds of the dose combinations present in the data (e.g., combinations of many face-to-face sessions and few maintenance phone calls). See Additional file 1 for complete distribution of dose received and additional file 5 for predicted estimates at each specific dose combination

The first dose interaction model (including both face-to-face and maintenance dose main effects and their interaction) demonstrated a significant interaction between face-to-face sessions and maintenance calls (B = -0.0059; 95% CI [− 0.0115, − 0.0004]; *p* = 0.037) for BMI-Z at 1-year. That is, the effect of the number of intervention sessions on child BMI-Z at 1-year differed according to the number of maintenance calls. As the contour plot of the model-based estimates from this analysis indicates, parent-child pairs who received higher numbers of *both* face-to-face intensive sessions (> 6) and maintenance calls (> 8) were predicted to have the lowest BMI-Z immediately following the 1-year intervention (Fig. 2). The second dose interaction model, predicting the odds of at least a 0.1 BMI-Z reduction immediately after the 1-year intervention, also demonstrated a significant dose modality interaction (B = 1.028; 95% CI [1.0018, 1.0540]; *p* = 0.036). Using a representative covariate profile, this model suggests that males with Hispanic Mexican parents, and the mean baseline BMI-Z and age have a predicted probability of 0.51 (95% CI [0.39, 0.63]) for a BMI-Z reduction of at least 0.1 when receiving the maximum dose in the first year. By contrast, this model predicted a probability of 0.27 (95% CI [0.15, 0.40]) for children with the same covariate profile who received only 4 face-to-face intensive sessions and 3 maintenance phone call sessions (Fig. 3).

In sensitivity analyses limiting the analytic sample to participants randomized to the intervention (*n* = 274), the directions of the dose-outcome coefficient point estimates were consistent with the above results (Additional file 5). Only the face-to-face dose analysis had a slightly attenuated point estimate, while the point estimates in the remaining analyses were either not affected, or, in the maintenance calls model, the estimate increased in magnitude. *P*-values for some, but not all, of the results from these sensitivity analyses were higher than those for the main analyses.

### Predictors of dose delivered

The adjusted linear regression model predicting the intensive weekly face-to-face dose delivered (Table 3) demonstrated significant associations for the non-Mexican Hispanic group versus the Mexican Hispanic reference group (B = -1.797; 95% CI [− 2.839, − 0.754]; *p* = 0.001) and for baseline child HEI (B = 0.041; 95% CI [0.005, 0.078]; *p* = 0.027). In the corresponding model predicting maintenance dose delivered, significant associations were found for female child versus male child (B = -0.610; 95% CI [− 1.218, − 0.002]; *p* = 0.049) and for baseline child HEI score (B = 0.030; 95% CI [0.003, 0.058]; *p* = 0.030).

**Table 3:** Sociodemographic characteristics predicting the amount of dose delivered. Results represent two separate multivariable linear regression models^a^

Predictor	Face-to-face modality			Maintenance modality		
	B	95% CI	*p*-value	B	95% CI	*p*-value
Baseline child age	−0.312	[− 0.754, 0.131]	0.166	− 0.088	[− 0.384, 0.208]	0.559
Baseline parent age	−0.030	[− 0.104, 0.044]	0.426	0.002	[−0.044, 0.048]	0.937
Child female (ref: male)	−0.271	[−1.149, 0.608]	0.544	−0.610	[−1.218, − 0.002]	0.049
Parent Hispanic non-Mexican (ref: Hispanic Mexican)	−1.797	[−2.839, − 0.754]	0.001	− 0.397	[− 1.091, 0.298]	0.262
Parent non-Hispanic (ref: Hispanic Mexican)	0.341	[−1.202, 1.885]	0.664	−0.726	[−2.056, 0.604]	0.283
Baseline child BMI-Z	0.207	[−0.754, 1.168]	0.672	0.568	[−0.066, 1.202]	0.079
Baseline child HEI	0.041	[0.005, 0.078]	0.027	0.030	[0.003, 0.058]	0.030
Baseline child % MVPA	−0.092	[− 0.257, 0.073]	0.273	0.031	[−0.057, 0.119]	0.487
WIC and/or SNAP use (ref: use neither)	−0.745	[−1.885, 0.394]	0.199	−0.323	[− 1.099, 0.452]	0.412
Parent depression (CES-D)	0.024	[−0.034, 0.082]	0.422	0.025	[−0.012, 0.062]	0.18
Parent Stress (PSS)	−0.017	[−0.108, 0.075]	0.719	−0.009	[− 0.066, 0.047]	0.745
Energy to change nutrition	−0.003	[−0.202, 0.196]	0.975	0.038	[−0.075, 0.150]	0.511
Energy to change physical activity	−0.032	[−0.194, 0.131]	0.702	−0.024	[− 0.120, 0.072]	0.620
Confidence: healthy growth	−0.077	[−0.326, 0.173]	0.545	−0.026	[− 0.206, 0.153]	0.772
Confidence: change eating	0.023	[−0.231, 0.276]	0.86	−0.015	[−0.210, 0.181]	0.881
Confidence: change physical activity	−0.077	[−0.347, 0.193]	0.574	−0.028	[− 0.263, 0.207]	0.817
Confidence: change media use	0.191	[−0.025, 0.407]	0.083	0.054	[−0.069, 0.177]	0.389
Parent classification of child weight	0.764	[−0.327, 1.856]	0.169	−0.203	[−1.003, 0.598]	0.619
Parent education: high school or further (ref: not completed high school)	−0.290	[−1.185, 0.604]	0.523	−0.252	[−0.851, 0.347]	0.408
Parent obese (ref: not obese)	−0.254	[−1.169, 0.660]	0.584	0.282	[−0.274, 0.839]	0.319

^a^*n* = 288 out of 304 intervention participants

## Discussion

This post-hoc and exploratory analysis of the Growing Right Onto Wellness trial suggests that a combination of intensive face-to-face sessions along with a monthly phone call “maintenance dose” is associated with lowest BMI-Z immediately after the 1 year intervention. Results indicate that a relatively small initial dose of 5–6 h of face-to-face contact over 2–3 months followed by 7–9 months of maintenance phone calls was associated with the lowest overall BMI-Z and increased probability of obtaining a BMI-Z reduction of at least 0.1 at 1-year. There was no statistically significant association between dose received of the intervention and BMI-Z at 2- or 3- year follow-up. Because of the exploratory nature of the analyses, self-selection of dose received, and relative sparsity of participants with lower dose ranges, the results should be interpreted with caution, replicated in other samples, and serve as hypothesis-generating for future randomized studies to prospectively evaluate the effect of dose of a behavioral intervention on BMI outcomes in children.

To our knowledge, this type of secondary “dose” analysis is a novel contribution in the field of behavioral obesity interventions. A meta-analysis of 20 studies by Janicke et al. found the dose of comprehensive behavioral family lifestyle interventions in the community or in outpatient clinical settings was associated with their efficacy at supporting healthy childhood growth [28]. In addition, one randomized controlled trial published in 2017 specifically tested a high dose intervention (32 h) versus a low dose intervention (8 h) to gauge *maintenance* of weight loss after a family-centered obesity intervention. This RCT did find a dose-response where the high dose maintenance condition was superior to the low dose group [29]. Our data add to this literature by suggesting that there may be specific combinations of the dose of a behavioral intervention that may cause differential improvements in child weight. In addition, our data suggest that there is a dose-response relationship, though the minimum number of contact hours may be less than the 26 h that has become the standard practice.

The challenge for researchers and policy makers to identify the optimal dose for obesity prevention and treatment remains. Our analyses indicate that clinically meaningful BMI-Z reduction in the context of childhood obesity prevention for underserved preschool aged children may be attained with fewer than 26 h (recommended by USPSTF for obesity treatment). However, the specific combination of contact hours, duration, and different modalities needs further study to identify the optimal approach to childhood obesity prevention in this population. In addition, sustained associations between the active intervention dose and outcome were not statistically significant at 2- or 3-year follow-up. One possible explanation is the increased variability in BMI-Z at 2 and 3 years. Replication with a larger sample size might provide the precision necessary to detect a potential effect, and/or identify subgroups with a stronger dose-outcome relationship. One implication of these findings is the need to test a longer active dose of a behavioral intervention to achieve maintenance of weight changes, especially among underserved populations at higher risk for childhood obesity.

We suggest that the methodology applied in this analysis advances the typical evaluation of a behavioral RCT. Unlike drug trials where the same dose of the intervention can consistently be given to participants, a behavioral intervention can have different amounts of “dose delivered” for each participant. Consequently, evaluation methods that focus on an “all-or-none” approach to effectiveness may be overlooking clinically meaningful impact for individuals who received the appropriate dose for them. This consideration is especially salient for under-represented, minority communities, where poverty and other socioeconomic hardships can prevent regular participation in behavioral trials. Consequently, determining how much of the intervention dose is necessary for which participants may be an important adjunct evaluation methodology that will have the capacity to reduce health disparities. Whereas the primary, intention-to-treat analysis for GROW indicated that the trial was not successful at affecting child BMI trajectories over 3-years, this analysis indicated that receiving all of the behavioral intervention dose throughout the first year was associated with a greater than 50% probability for a clinically significant BMI-Z reduction immediately following the 1-year intervention. Simply using an “all-or-none” approach would have obscured this clinically meaningful result.

The study had several limitations. The major limitation to this analytic framework is the potential for confounding: the idea that there may be certain characteristics of individuals who are more likely to attend sessions that also make them more likely to be successful at behavior change and obesity prevention. Receipt of a higher intervention dose is not random, and it was not manipulated in the current study’s experimental design. Therefore, it is important to consider predictors of intervention exposure when assessing intervention efficacy [30]. We attempted to account for this in the current analysis by predicting the dose delivered from important baseline sociodemographic variables, including parent confidence in ability to change. However, causality between dose and the outcomes cannot be confirmed. Another limitation is the relative sparseness of the data at low intervention doses (particularly for the maintenance dose). Because the trial had high dose delivery, model estimates for certain combinations of low-intensity intervention doses are based on limited information. Consequently, estimated results at these combinations should be interpreted with caution. We included in the main model participants from the control group, who had an intervention dose of zero. We also conducted sensitivity analyses limiting the original models to participants randomized only to the intervention group. The sensitivity analyses should be interpreted with caution given the reduction in sample size (by half) compared to the overall model. We suggest that the main result from the current analyses should not be a firm conclusion of how much dose is needed for childhood obesity prevention. Rather, this should serve as the basis for generating new testable hypotheses based on dose frequency, type, and duration.

## Conclusion

In conclusion, the findings from this trial of a behavioral preventive intervention for childhood obesity suggest that young underserved children can experience clinically meaningful improvement in BMI outcomes over 1 year with a multi-modal dose delivery that is less than 26 h. Because these changes in BMI were not significantly sustained at 2- or 3-year follow-up, additional investigation into the best interventions of maintenance of weight loss remain an important step.

## Supplementary information

**Additional file 1.** Distribution of Intensive Face-to-Face and Maintenance Dose.

**Additional file 2.** Predicting BMI-Z immediately following the 1-year intervention in using three separate adjusted linear regression models with the following predictors: Model 1) face-to-face intensive modality; Model 2) maintenance phone call modality; and Model 3) modality main effects and interaction. Each model controls for child age, child gender, parent race/ethnicity, and baseline child BMI-Z.

**Additional file 3.** Results predicting BMI-Z at 2- and 3-year follow-up in three separate adjusted linear regression models using 1) face-to-face intensive modality 2) maintenance phone call modality, and 3) modality main effects and interaction.

**Additional file 4.** Model-based estimates for each specific combination of dose received, predicting child BMI-Z score immediately following the 1-year intervention and the probability of at least 0.1 probability of at least a 0.1 decrease in BMI-Z immediately following the 1-year intervention.

**Additional file 5.** Sensitivity Analysis of the main analytic model, excluding control group participants.

## Data Availability

The datasets generated during and/or analyzed during the current study will be made available to the public no later than 3 years after the end of NIH support.

## Abbreviations

USPSTF: U.S. Preventive Services Task Force
NIH: National Institutes of Health
BMI-Z: Body Mass Index Z-Score
GROW: Growing Right Onto Wellness
RCT: Randomized Controlled Trial
BMI: Body Mass Index
IRB: Institutional Review Board
CDC: Centers for Disease Control and Prevention
HEI: Health Eating Index
MVPA: Moderate and Vigorous Physical Activity
SNAP: Supplemental Nutrition Assistance Program
WIC: The Special Supplemental Nutrition Program for Women, Infants, and Children
