# Supplementary material for: Evaluating dose delivered of a behavioral intervention for childhood obesity prevention: a secondary analysis
Source: BMC Public Health. 2020 Jun 8;20:885. doi: 10.1186/s12889-020-09020-w (PMC7281919; doi:10.1186/s12889-020-09020-w)
Supplement: Supplementary file 1 — Additional file 1. Distribution of Intensive Face-to-Face and Maintenance Dose. [file 12889_2020_9020_MOESM1_ESM.docx]

|  | **Additional File 1: Distribution of Intensive Face-to-Face and Maintenance Dose** | | | | | | | | | | | | |
| --- | --- | --- | --- | --- | --- | --- | --- | --- | --- | --- | --- | --- | --- |
|  | |  | | **# of Maintenance Calls** | | | | | | | | | |
| **# Face-to-Face Intensive Sessions** | | 0 | 1 | | 2 | 3 | 4 | 5 | 6 | 7 | 8 | 9 | **Row Total** |
| 12 | | 0 | 0 | | 0 | 0 | 0 | 0 | 0 | 0 | 5 | 18 | 23 |
| 11 | | 0 | 0 | | 0 | 1 | 0 | 0 | 0 | 1 | 4 | 32 | 38 |
| 10 | | 0 | 0 | | 1 | 0 | 1 | 1 | 0 | 4 | 10 | 28 | 45 |
| 9 | | 1 | 1 | | 0 | 1 | 0 | 1 | 2 | 2 | 4 | 24 | 36 |
| 8 | | 0 | 0 | | 0 | 0 | 0 | 0 | 1 | 1 | 4 | 19 | 25 |
| 7 | | 0 | 0 | | 0 | 0 | 0 | 1 | 0 | 2 | 3 | 15 | 21 |
| 6 | | 2 | 1 | | 1 | 1 | 3 | 2 | 3 | 3 | 3 | 7 | 26 |
| 5 | | 0 | 0 | | 1 | 0 | 1 | 0 | 1 | 3 | 1 | 7 | 14 |
| 4 | | 1 | 0 | | 1 | 0 | 0 | 0 | 1 | 0 | 2 | 6 | 11 |
| 3 | | 0 | 0 | | 0 | 0 | 1 | 2 | 0 | 1 | 2 | 8 | 14 |
| 2 | | 0 | 1 | | 0 | 0 | 0 | 0 | 0 | 3 | 4 | 8 | 16 |
| 1 | | 4 | 1 | | 1 | 1 | 1 | 1 | 2 | 1 | 1 | 4 | 17 |
| 0 | | 4 | 1 | | 0 | 0 | 0 | 1 | 2 | 0 | 3 | 7 | 18 |
| **Column Total** | | 12 | 5 | | 5 | 4 | 7 | 9 | 12 | 21 | 46 | 183 | 304 |
|  | Cells represent the number of individuals who received each combination of face-to-face intensive and maintenance calls. There were 304 Individuals randomized to the intervention. | | | | | | | | | | | | |
