# Supplementary material for: Evaluating dose delivered of a behavioral intervention for childhood obesity prevention: a secondary analysis
Source: BMC Public Health. 2020 Jun 8;20:885. doi: 10.1186/s12889-020-09020-w (PMC7281919; doi:10.1186/s12889-020-09020-w)
Supplement: Supplementary file 2 — Additional file 2. Predicting BMI-Z immediately following the 1-year intervention in using three separate adjusted linear regression models with the following predictors: Model 1) face-to-face intensive modality; Model 2) maintenance phone call modality; and Model 3) modality main effects and interaction. Each model controls for child age, child gender, parent race/ethnicity, and baseline child BMI-Z. [file 12889_2020_9020_MOESM2_ESM.docx]

**Additional File 2: Predicting BMI-Z at 1-year follow-up in using three separate adjusted linear regression models with the following predictors: Model 1) face-to-face intensive modality; Model 2) maintenance phone call modality; and Model 3) modality main effects and interaction. Each model controls for child age, child gender, parent race/ethnicity, and baseline child BMI-Z.**

|  | **Year 1** | n=549 |  |
| --- | --- | --- | --- |
| **Model 1: face-to-face intensive modality** | Regression coefficient |  |  |
| Baseline child BMI-Z | 0·935 | [0·838, 1·033] | <0·001 |
| Face-to-face dose | -0·011 | [-0·021, -0·001] | 0·029 |
| Child female (ref: male) | -0·035 | [-0·127, 0·056] | 0·449 |
| Baseline child age | 0·100 | [0·048, 0·152] | <0·001 |
| Parent Hispanic non-Mexican (ref: Hispanic Mexican) | -0·035 | [-0·140, 0·069] | 0·507 |
| Parent non-Hispanic (ref: Hispanic Mexican) | -0·075 | [-0·238, 0·088] | 0·363 |
| **Model 2: maintenance phone call modality** |  |  |  |
| Baseline child BMI-Z | 0·940 | [0·843, 1·037] | <0·001 |
| Maintenance dose | -0·015 | [-0·026, -0·004] | 0·006 |
| Child female (ref: male) | -0·039 | [-0·131 ,0·052] | 0·398 |
| Baseline child age | 0·101 | [0·048, 0·153] | <0·001 |
| Parent Hispanic non-Mexican (ref: Hispanic Mexican) | -0·025 | [-0·130, 0·079] | 0·635 |
| Parent non-Hispanic (ref: Hispanic Mexican) | -0·083 | [-0·245, 0·080] | 0·319 |
| **Model 3: modality main effects and interaction** |  |  |  |
| Baseline child BMI-Z | 0·945 | [0·847, 1·042] | <0·001 |
| Face-to-face dose | 0·046 | [0·001, 0·091] | 0·044 |
| Maintenance dose | -0·010 | [-0·031, 0·012] | 0·385 |
| Interaction: Face-to-face by maintenance | -0·0059 | [-0·0115, -0·0004] | 0·037 |
| Child female (ref: male) | -0·042 | [-0·133, 0·049] | 0·369 |
| Baseline child age | 0·099 | [0·047, 0·151] | <0·001 |
| Parent Hispanic non-Mexican (ref: Hispanic Mexican) | -0·030 | [-0·135, 0·075] | 0·578 |
| Parent non-Hispanic (ref: Hispanic Mexican) | -0·097 | [-0·260, 0·066] | 0·244 |
