# Supplementary material for: Evaluating dose delivered of a behavioral intervention for childhood obesity prevention: a secondary analysis
Source: BMC Public Health. 2020 Jun 8;20:885. doi: 10.1186/s12889-020-09020-w (PMC7281919; doi:10.1186/s12889-020-09020-w)
Supplement: Supplementary file 3 — Additional file 3. Results predicting BMI-Z at 2- and 3-year follow-up in three separate adjusted linear regression models using 1) face-to-face intensive modality 2) maintenance phone call modality, and 3) modality main effects and interaction. [file 12889_2020_9020_MOESM3_ESM.docx]

**Additional File 3:** Results predicting BMI-Z at 2- and 3-year follow-up in three separate adjusted linear regression models using 1) face-to-face intensive modality 2) maintenance phone call modality, and 3) modality main effects and interaction

|  | **2 years** | n=544 |  | **3 years** | n=548 |  |
| --- | --- | --- | --- | --- | --- | --- |
| **Model 1: face-to-face intensive modality** | Regression coefficient | 95% CI | p-value | Regression coefficient | 95% CI | p-value |
| Baseline child BMI-Z | 1.007 | [0.884, 1.131] | <0.001 | 1.049 | [0.898, 1.200] | <0.001 |
| Face-to-face dose | -0.008 | [-0.021, 0.005] | 0.231 | -0.009 | [-0.025, 0.006] | 0.240 |
| Child female (ref: male) | -0.013 | [-0.129, 0.102] | 0.824 | -0.139 | [-0.281, 0.002] | 0.054 |
| Baseline child age | 0.215 | [0.149, 0.280] | <0.001 | 0.323 | [0.243, 0.404] | <0.001 |
| Parent Hispanic non-Mexican (ref: Hispanic Mexican) | -0.003 | [-0.137, 0.131] | 0.967 | 0.007 | [-0.155, 0.170] | 0.931 |
| Parent non-Hispanic (ref: Hispanic Mexican) | -0.030 | [-0.237, 0.176] | 0.773 | -0.003 | [-0.259, 0.253] | 0.982 |
| **Model 2: maintenance phone call modality** |  |  |  |  |  |  |
| Baseline child BMI-Z | 1.008 | [0.884, 1.132] | <0.001 | 1.051 | [0.900, 1.202] | <0.001 |
| Maintenance dose | -0.006 | [-0.020, 0.008] | 0.385 | -0.010 | [-0.026, 0.007] | 0.266 |
| Child female (ref: male) | -0.014 | [-0.130, 0.102] | 0.814 | -0.140 | [-0.282, 0.001] | 0.052 |
| Baseline child age | 0.216 | [0.151, 0.282] | <0.001 | 0.325 | [0.244, 0.405] | <0.001 |
| Parent Hispanic non-Mexican (ref: Hispanic Mexican) | 0.002 | [-0.132, 0.136] | 0.981 | 0.014 | [-0.148, 0.177] | 0.862 |
| Parent non-Hispanic (ref: Hispanic Mexican) | -0.031 | [-0.239, 0.176] | 0.766 | -0.004 | [-0.260, 0.253] | 0.976 |
| **Model 3: modality main effects and interaction** |  |  |  |  |  |  |
| Baseline child BMI-Z | 1.013 | [0.890, 1.137] | <0.001 | 1.057 | [0.906, 1.208] | <0.001 |
| Face-to-face dose | 0.045 | [-0.011, 0.100] | 0.114 | 0.053 | [-0.022, 0.128] | 0.165 |
| Maintenance dose | 0.015 | [-0.013, 0.043] | 0.289 | 0.006 | [-0.028, 0.041] | 0.716 |
| Interaction: Face-to-face by maintenance | -0.008 | [-0.014, -0.001] | 0.029 | -0.008 | [-0.017, 0.001] | 0.087 |
| Child female (ref: male) | -0.014 | [-0.130, 0.101] | 0.807 | -0.145 | [-0.286, -0.003] | 0.045 |
| Baseline child age | 0.213 | [0.147, 0.279] | <0.001 | 0.324 | [0.244, 0.405] | <0.001 |
| Parent Hispanic non-Mexican (ref: Hispanic Mexican) | -0.011 | [-0.146, 0.123] | 0.867 | 0.006 | [-0.159, 0.170] | 0.947 |
| Parent non-Hispanic (ref: Hispanic Mexican) | -0.039 | [-0.245, 0.168] | 0.714 | -0.011 | [-0.267, 0.246] | 0.936 |
