# Supplementary material for: Evaluating dose delivered of a behavioral intervention for childhood obesity prevention: a secondary analysis
Source: BMC Public Health. 2020 Jun 8;20:885. doi: 10.1186/s12889-020-09020-w (PMC7281919; doi:10.1186/s12889-020-09020-w)
Supplement: Supplementary file 4 — Additional file 4. Model-based estimates for each specific combination of dose received, predicting child BMI-Z score immediately following the 1-year intervention and the probability of at least 0.1 probability of at least a 0.1 decrease in BMI-Z immediately following the 1-year intervention. [file 12889_2020_9020_MOESM4_ESM.docx]

|  |  | Number of Maintenance Phone Calls Received | | | | | | | | | |
| --- | --- | --- | --- | --- | --- | --- | --- | --- | --- | --- | --- |
|  |  | 0 | 1 | 2 | 3 | 4 | 5 | 6 | 7 | 8 | 9 |
| Number of Intensive Face-to-Face Sessions Attended | 12 | 1.49 [0.95, 2.02] | 1.41 [0.93, 1.88] | 1.32 [0.91, 1.74] | 1.24 [0.89, 1.60] | 1.16 [0.86, 1.46] | 1.08 [0.84, 1.33] | 1.00 [0.81, 1.19] | 0.92 [0.77, 1.07] | 0.84 [0.72, 0.96] | 0.76 [0.64, 0.88] |
|  | 11 | 1.44 [0.95, 1.93] | 1.36 [0.93, 1.80] | 1.29 [0.91, 1.67] | 1.22 [0.89, 1.54] | 1.14 [0.87, 1.42] | 1.07 [0.84, 1.29] | 0.99 [0.82, 1.17] | 0.92 [0.78, 1.05] | 0.84 [0.73, 0.95] | 0.77 [0.66, 0.88] |
|  | 10 | 1.39 [0.94, 1.84] | 1.32 [0.93, 1.72] | 1.26 [0.91, 1.60] | 1.19 [0.89, 1.49] | 1.12 [0.87, 1.37] | 1.05 [0.85, 1.25] | 0.98 [0.82, 1.14] | 0.91 [0.79, 1.03] | 0.84 [0.74, 0.94] | 0.77 [0.67, 0.87] |
|  | 9 | 1.35 [0.94, 1.75] | 1.28 [0.92, 1.64] | 1.22 [0.91, 1.54] | 1.16 [0.89, 1.43] | 1.10 [0.87, 1.32] | 1.03 [0.85, 1.22] | 0.97 [0.83, 1.11] | 0.91 [0.80, 1.02] | 0.84 [0.75, 0.94] | 0.78 [0.69, 0.87] |
|  | 8 | 1.30 [0.94, 1.66] | 1.24 [0.92, 1.56] | 1.19 [0.91, 1.47] | 1.13 [0.89, 1.37] | 1.07 [0.87, 1.28] | 1.02 [0.85, 1.18] | 0.96 [0.83, 1.09] | 0.90 [0.80, 1.01] | 0.85 [0.76, 0.93] | 0.79 [0.70, 0.88] |
|  | 7 | 1.25 [0.94, 1.57] | 1.20 [0.92, 1.49] | 1.15 [0.91, 1.40] | 1.10 [0.89, 1.31] | 1.05 [0.87, 1.23] | 1.00 [0.85, 1.15] | 0.95 [0.83, 1.07] | 0.90 [0.80, 1.00] | 0.85 [0.76, 0.94] | 0.80 [0.70, 0.89] |
|  | 6 | 1.21 [0.93, 1.48] | 1.16 [0.92, 1.41] | 1.12 [0.90, 1.33] | 1.07 [0.89, 1.26] | 1.03 [0.87, 1.19] | 0.98 [0.85, 1.12] | 0.94 [0.83, 1.05] | 0.89 [0.80, 0.99] | 0.85 [0.75, 0.94] | 0.80 [0.70, 0.90] |
|  | 5 | 1.16 [0.93, 1.39] | 1.12 [0.92, 1.33] | 1.08 [0.90, 1.27] | 1.04 [0.89, 1.20] | 1.01 [0.87, 1.14] | 0.97 [0.85, 1.09] | 0.93 [0.82, 1.03] | 0.89 [0.79, 0.99] | 0.85 [0.75, 0.95] | 0.81 [0.70, 0.92] |
|  | 4 | 1.12 [0.93, 1.31] | 1.08 [0.91, 1.25] | 1.05 [0.90, 1.20] | 1.02 [0.88, 1.15] | 0.98 [0.86, 1.10] | 0.95 [0.84, 1.06] | 0.92 [0.81, 1.02] | 0.88 [0.78, 0.99] | 0.85 [0.74, 0.96] | 0.82 [0.69, 0.94] |
|  | 3 | 1.07 [0.92, 1.22] | 1.04 [0.91, 1.18] | 1.02 [0.89, 1.14] | 0.99 [0.87, 1.10] | 0.96 [0.85, 1.07] | 0.93 [0.83, 1.04] | 0.91 [0.80, 1.01] | 0.88 [0.76, 0.99] | 0.85 [0.73, 0.98] | 0.82 [0.68, 0.96] |
|  | 2 | 1.02 [0.91, 1.14] | 1.00 [0.90, 1.11] | 0.98 [0.88, 1.08] | 0.96 [0.86, 1.06] | 0.94 [0.84, 1.04] | 0.92 [0.81, 1.02] | 0.90 [0.78, 1.01] | 0.87 [0.75, 1.00] | 0.85 [0.71, 0.99] | 0.83 [0.67, 0.99] |
|  | 1 | 0.98 [0.89, 1.07] | 0.96 [0.88, 1.05] | 0.95 [0.86, 1.03] | 0.93 [0.84, 1.02] | 0.92 [0.82, 1.01] | 0.90 [0.79, 1.01] | 0.88 [0.76, 1.01] | 0.87 [0.73, 1.01] | 0.85 [0.70, 1.01] | 0.84 [0.66, 1.01] |
|  | 0 | 0.93 [0.85, 1.02] | 0.92 [0.84, 1.00] | 0.91 [0.83, 1.00] | 0.90 [0.81, 1.00] | 0.89 [0.79, 1.00] | 0.88 [0.76, 1.00] | 0.87 [0.74, 1.01] | 0.86 [0.71, 1.02] | 0.85 [0.68, 1.03] | 0.85 [0.65, 1.04] |

Full model-based estimates of child BMI-Z score immediately following the 1-year intervention.

Full model-based estimates of the probability of at least a 0.1 decrease in BMI-Z immediately following the 1-year intervention.

|  |  | Number of Maintenance Phone Calls Received | | | | | | | | | |
| --- | --- | --- | --- | --- | --- | --- | --- | --- | --- | --- | --- |
|  |  | 0 | 1 | 2 | 3 | 4 | 5 | 6 | 7 | 8 | 9 |
| Number of Intensive Face-to-Face Sessions Attended | 12 | 0.05 [-0.07, 0.18] | 0.07 [-0.08, 0.22] | 0.10 [-0.08, 0.27] | 0.13 [-0.06, 0.32] | 0.17 [-0.03, 0.37] | 0.22 [0.02, 0.41] | 0.28 [0.10, 0.46] | 0.35 [0.20, 0.50] | 0.43 [0.31, 0.54] | 0.51 [0.39, 0.63] |
|  | 11 | 0.06 [-0.08, 0.20] | 0.08 [-0.08, 0.24] | 0.11 [-0.07, 0.28] | 0.14 [-0.05, 0.33] | 0.18 [-0.01, 0.37] | 0.23 [0.05, 0.41] | 0.29 [0.12, 0.45] | 0.35 [0.22, 0.48] | 0.42 [0.31, 0.53] | 0.49 [0.39, 0.60] |
|  | 10 | 0.08 [-0.07, 0.23] | 0.10 [-0.07, 0.26] | 0.12 [-0.06, 0.30] | 0.16 [-0.03, 0.34] | 0.20 [0.01, 0.38] | 0.24 [0.07, 0.41] | 0.29 [0.14, 0.45] | 0.35 [0.23, 0.47] | 0.42 [0.32, 0.51] | 0.48 [0.39, 0.58] |
|  | 9 | 0.09 [-0.07, 0.25] | 0.11 [-0.06, 0.29] | 0.14 [-0.04, 0.32] | 0.17 [-0.01, 0.35] | 0.21 [0.03, 0.39] | 0.25 [0.09, 0.41] | 0.30 [0.16, 0.44] | 0.35 [0.24, 0.47] | 0.41 [0.32, 0.50] | 0.47 [0.38, 0.56] |
|  | 8 | 0.11 [-0.06, 0.28] | 0.13 [-0.04, 0.31] | 0.16 [-0.02, 0.34] | 0.19 [0.02, 0.36] | 0.23 [0.06, 0.39] | 0.27 [0.12, 0.41] | 0.31 [0.18, 0.44] | 0.36 [0.25, 0.46] | 0.41 [0.32, 0.49] | 0.46 [0.37, 0.55] |
|  | 7 | 0.13 [-0.04, 0.30] | 0.15 [-0.02, 0.33] | 0.18 [0.01, 0.35] | 0.21 [0.04, 0.37] | 0.24 [0.09, 0.40] | 0.28 [0.14, 0.41] | 0.32 [0.20, 0.43] | 0.36 [0.26, 0.46] | 0.40 [0.32, 0.49] | 0.45 [0.36, 0.54] |
|  | 6 | 0.15 [-0.01, 0.32] | 0.18 [0.01, 0.34] | 0.20 [0.04, 0.36] | 0.23 [0.08, 0.38] | 0.26 [0.12, 0.40] | 0.29 [0.17, 0.42] | 0.33 [0.22, 0.43] | 0.36 [0.27, 0.45] | 0.40 [0.31, 0.49] | 0.44 [0.34, 0.53] |
|  | 5 | 0.18 [0.02, 0.35] | 0.20 [0.05, 0.36] | 0.23 [0.08, 0.38] | 0.25 [0.11, 0.39] | 0.28 [0.15, 0.40] | 0.30 [0.19, 0.42] | 0.33 [0.23, 0.43] | 0.36 [0.27, 0.46] | 0.40 [0.30, 0.49] | 0.43 [0.32, 0.53] |
|  | 4 | 0.21 [0.06, 0.36] | 0.23 [0.09, 0.37] | 0.25 [0.12, 0.39] | 0.27 [0.15, 0.40] | 0.30 [0.18, 0.41] | 0.32 [0.21, 0.42] | 0.34 [0.24, 0.44] | 0.37 [0.27, 0.46] | 0.39 [0.29, 0.50] | 0.42 [0.30, 0.53] |
|  | 3 | 0.25 [0.12, 0.38] | 0.27 [0.14, 0.39] | 0.28 [0.17, 0.39] | 0.30 [0.19, 0.40] | 0.31 [0.21, 0.41] | 0.33 [0.23, 0.43] | 0.35 [0.25, 0.45] | 0.37 [0.26, 0.47] | 0.39 [0.27, 0.50] | 0.41 [0.28, 0.54] |
|  | 2 | 0.29 [0.18, 0.40] | 0.30 [0.20, 0.40] | 0.31 [0.22, 0.40] | 0.32 [0.23, 0.41] | 0.33 [0.24, 0.43] | 0.35 [0.25, 0.44] | 0.36 [0.25, 0.46] | 0.37 [0.26, 0.48] | 0.38 [0.25, 0.51] | 0.40 [0.25, 0.54] |
|  | 1 | 0.33 [0.25, 0.42] | 0.34 [0.26, 0.42] | 0.34 [0.26, 0.42] | 0.35 [0.27, 0.43] | 0.35 [0.27, 0.44] | 0.36 [0.26, 0.46] | 0.37 [0.26, 0.48] | 0.37 [0.25, 0.50] | 0.38 [0.24, 0.52] | 0.38 [0.23, 0.54] |
|  | 0 | 0.38 [0.30, 0.46] | 0.38 [0.30, 0.45] | 0.38 [0.30, 0.45] | 0.38 [0.29, 0.46] | 0.38 [0.28, 0.47] | 0.38 [0.27, 0.48] | 0.38 [0.25, 0.50] | 0.37 [0.24, 0.51] | 0.37 [0.22, 0.53] | 0.37 [0.20, 0.55] |
