# Supplementary material for: Evaluating dose delivered of a behavioral intervention for childhood obesity prevention: a secondary analysis
Source: BMC Public Health. 2020 Jun 8;20:885. doi: 10.1186/s12889-020-09020-w (PMC7281919; doi:10.1186/s12889-020-09020-w)
Supplement: Supplementary file 5 — Additional file 5. Sensitivity Analysis of the main analytic model, excluding control group participants. [file 12889_2020_9020_MOESM5_ESM.docx]

**Additional File 5: Sensitivity analysis excluding control group and predicting BMI-Z at 1-year follow-up in using three separate adjusted linear regression models with the following predictors: Model 1) face-to-face intensive modality; Model 2) maintenance phone call modality; and Model 3) modality main effects and interaction. Each model controls for child age, child gender, parent race/ethnicity, and baseline child BMI-Z.**

|  | **Year 1** | n=274 |  |
| --- | --- | --- | --- |
| **Model 1: face-to-face intensive modality** | Regression coefficient |  |  |
| Baseline child BMI-Z | 0.936 | [0.802, 1.071] | <0.001 |
| Face-to-face dose | -0.008 | [-0.027, 0.010] | 0.370 |
| Child female (ref: male) | -0.029 | [-0.156, 0.098] | 0.654 |
| Baseline child age | 0.159 | [0.088, 0.231] | <0.001 |
| Parent Hispanic non-Mexican (ref: Hispanic Mexican) | -0.084 | [-0.228, 0.060] | 0.251 |
| Parent non-Hispanic (ref: Hispanic Mexican) | -0.136 | [-0.376, 0.103] | 0.264 |
| **Model 2: maintenance phone call modality** |  |  |  |
| Baseline child BMI-Z | 0.959 | [0.825, 1.093] | <0.001 |
| Maintenance dose | -0.043 | [-0.076, -0.010] | 0.010 |
| Child female (ref: male) | -0.054 | [-0.181, 0.074] | 0.408 |
| Baseline child age | 0.158 | [0.087, 0.228] | <0.000 |
| Parent Hispanic non-Mexican (ref: Hispanic Mexican) | -0.078 | [-0.218, 0.062] | 0.276 |
| Parent non-Hispanic (ref: Hispanic Mexican) | -0.166 | [-0.404, 0.072] | 0.172 |
| **Model 3: modality main effects and interaction** |  |  |  |
| Baseline child BMI-Z | 0.953 | [0.818, 1.088] | <0.001 |
| Face-to-face dose | 0.045 | [-0.028, 0.118] | 0.227 |
| Maintenance dose | -0.011 | [-0.070, 0.047] | 0.702 |
| Interaction: Face-to-face by maintenance | -0.006 | [-0.015, 0.003] | 0.200 |
| Child female (ref: male) | -0.042 | [-0.171, 0.086] | 0.517 |
| Baseline child age | 0.158 | [0.087, 0.229] | <0.001 |
| Parent Hispanic non-Mexican (ref: Hispanic Mexican) | -0.079 | [-0.222, 0.063] | 0.274 |
| Parent non-Hispanic (ref: Hispanic Mexican) | -0.180 | [-0.420, 0.059] | 0.140 |
